# A possible instance of sexual dimorphism in the tails of two oviraptorosaur dinosaurs

**DOI:** 10.1038/srep09472

**Published:** 2015-03-31

**Authors:** W. Scott Persons IV, Gregory F. Funston, Philip J. Currie, Mark A. Norell

**Affiliations:** 1 University of Alberta, Department of Biological Sciences, Edmonton, Alberta, T6G2E9, Canada; 2 American Museum of Natural History, New York City, New York, USA

## Abstract

The hypothesis that oviraptorosaurs used tail-feather displays in courtship behavior previously predicted that oviraptorosaurs would be found to display sexually dimorphic caudal osteology. MPC-D 100/1002 and MPC-D 100/1127 are two specimens of the oviraptorosaur *Khaan mckennai*. Although similar in absolute size and in virtually all other anatomical details, the anterior haemal spines of MPC-D 100/1002 exceed those of MPC-D 100/1127 in ventral depth and develop a hitherto unreported “spearhead” shape. This dissimilarity cannot be readily explained as pathologic and is too extreme to be reasonably attributed to the amount of individual variation expected among con-specifics. Instead, this discrepancy in haemal spine morphology may be attributable to sexual dimorphism. The haemal spine form of MPC-D 100/1002 offers greater surface area for caudal muscle insertions. On this basis, MPC-D 100/1002 is regarded as most probably male, and MPC-D 100/1127 is regarded as most probably female.

As in all major vertebrate groups, dinosaurs must have included many species with gross anatomical traits that were sexually dimorphic. However, the identification of sexual dimorphism in dinosaurs is hindered by the limitations of an ancient fossil record, which restricts comparative sample size, degrades the quality of available specimens, and usually precludes the observation of non-osteological features. Among non-avian theropod dinosaurs, previous attempts to recognize sexual dimorphism have been controversial and inconclusive. Here we describe a possible instance of sexual dimorphism based on chevron morphology in the oviraptorosaur *Khaan mckennai*.

Two specimens of *Khaan mckennai* (Paleontological Center of the Mongolian Academy of Sciences MPC-D 100/1002 and MPC-D 100/1127) were excavated from the same Upper Cretaceous locality in the Djadokhta Formation (Ukhaa Tolgod, Gurvan Tes Somon, Omnogov Aimak, Gobi Desert, Mongolia), and were found in close proximity to each other (approximately 20 cm away) and in the same bedding plane^1^. Geological work at Ukhaa Tolgod indicates that the preserved animals were buried alive by catastrophic dune collapses, precipitated by heavy rains^2^,^3^. MPC-D 100/1002 and MPC-D 100/1127 were likely killed by a single collapse event and appear to have been near each other prior to death. The two specimens were collectively given the informal, but perhaps fortuitous, nicknames of “Romeo and Juliet” or, occasionally, “Sid and Nancy”.

MPC-D 100/1002 is slightly larger than MPC-D 100/1127 (femur lengths of 195 mm and 190 mm, respectively). MPC-D 100/1127 – the holotype of *Khaan mckennai*^1^ – is a complete skeleton. MPC-D 100/1002 is a nearly complete skeleton, missing only the middle and posterior portions of the tail. It nevertheless displays all diagnostic characteristics of *Khaan mckennai*, including a proximally narrow metacarpal III that does not contact the distal carpals (for a full description of both specimens and their taxonomic assignment see Clark et al.^1^ and Balanoff and Norell^4^). In both individuals, all vertebral neural arches and centra are fully fused, indicating that both had reached adulthood before death^5^,^6^, although histological evidence is still wanting. Based on the similarities in size and proportions, both individuals also appear to have reached roughly the same level of maturity. However, the chevrons of MPC-D 100/1002 and MPC-D 100/1127 show a striking disparity in morphology.

## Description

**Figure 1:**
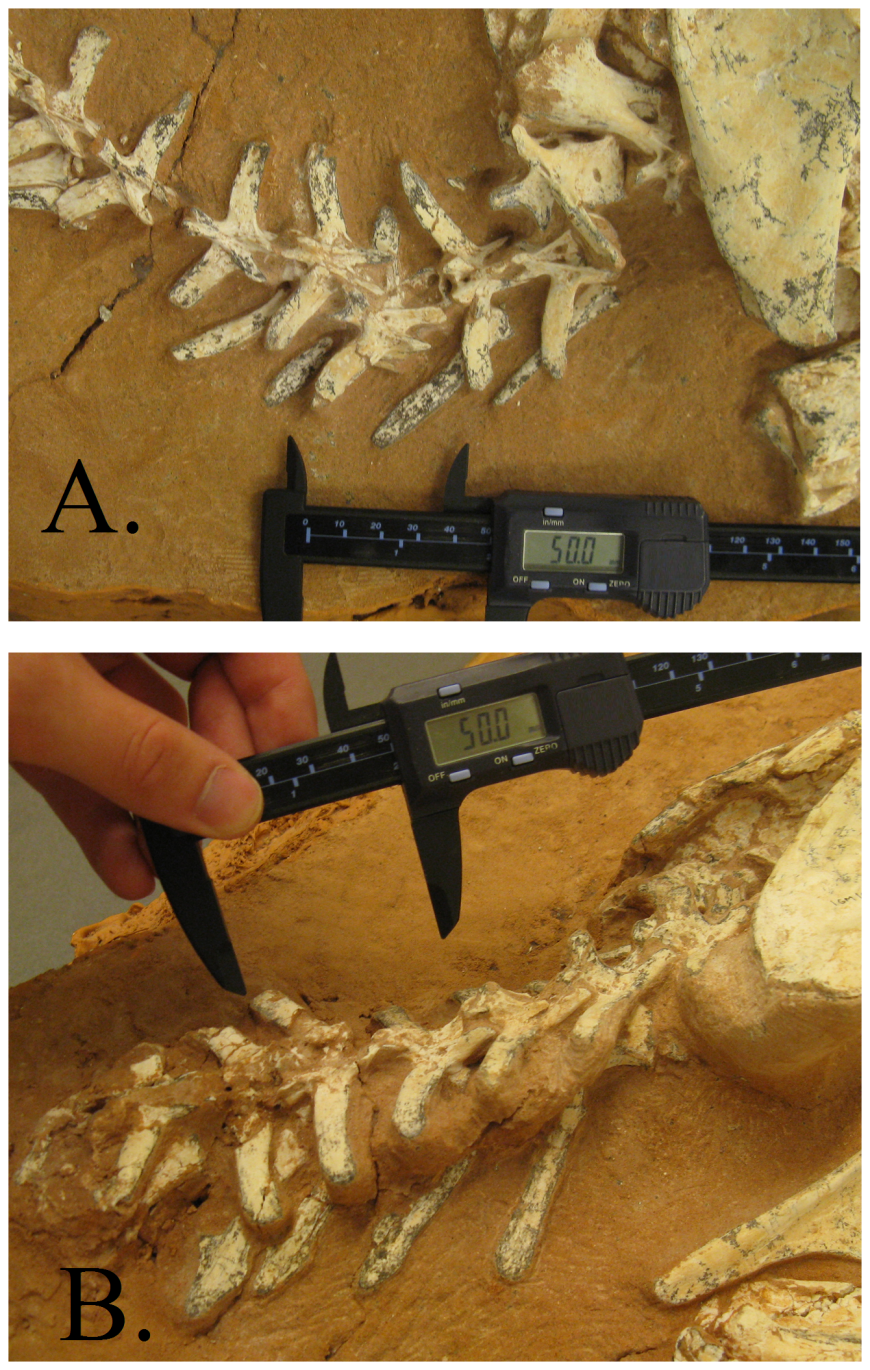
The anterior caudal sequence of MPC-D 100/1127 (A) and MPC-D 100/1002 (B).

**Figure 2:**
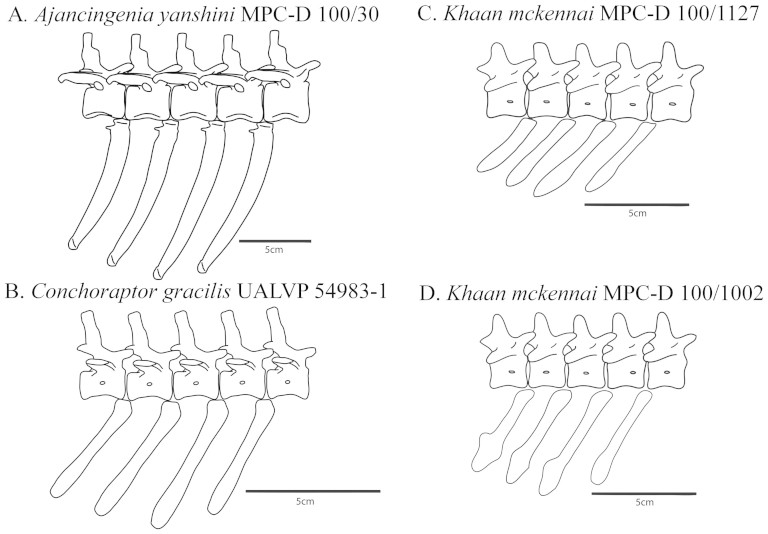
The first five caudal vertebrae and four chevrons of the oviraptorosaurs *Ajancingenia yanshini* MPC-D 100/30 (A), *Conchoraptor gracilis* UALVP 54983-1 (representative of the morphology observed in six additional specimens – see Supplemental Table 1) (B), *Khaan mckennai* MPC-D 100/1127 (C), and *Khaan mckennai* MPC-D 100/1002 (D) in right lateral view.

The anterior chevrons of MPC-D 100/1127 have an overall form that is typical of non-avian theropods, including that previously reported from other oviraptorosaurs (Figure 1). The anterior haemal spines are flat laterally and are simple finger-like projections. In terms of dorsoventral depth, the haemal spine of the second chevron is the deepest, and all more posterior haemal spines sequentially decrease in depth. At roughly the middle of the tail, the haemal spines gradually transition into a more boot-shaped form, with weak anterior projections and strong posterior projections (Figure 1 and 2). Although the posterior haemal spines of some oviraptorosaurs (such as *Anzu wyliei* CM 78000 and 78001, *Gigantoraptor erlianensis* LH V0011, and *Nomingia gobiensis* MPC-D 100/119) are rectangular^7^, the general pattern of gradual chevron shape change seen in MPC-D 100/1127 is typical, not only of oviraptorosaurs, but of most non-avian coelurosaurs^8^.

As in MPC-D 100/1127, the first chevron of MPC-D 100/1002 lies between the second and third caudal vertebrae; it also has a lateromedially flat haemal spine that is a simple finger-like projection, although the ventral tip is proportionately wider anteroposteriorly than that of the first chevron of MPC-D 100/1127. The second chevron of MPC-D 100/1002 has a haemal spine that is transversely flat and has a prominent posterior heel-like projection located roughly two thirds the way down the central shaft. The haemal spines of the third and fourth chevrons resemble that of the second, but are sequentially shorter, and have an increasingly more prominent posterior projection. Each also has a smaller, but increasingly prominent, anterior projection that is slightly ventral to the posterior projection. The haemal spine of the fourth chevron has a distinctive ventrally-projecting spear-head shape (Figure 1 and 2). Unfortunately, only the first four chevrons of MPC-D 100/1002 are preserved, and it is impossible to determine if the shape of the haemal spine form of the fourth chevron is representative of more posterior chevrons, or if the haemal spine shape changed posteriorly. Relative to the proportions of the vertebrae, the anterior haemal spines of MPC-D 100/1002 all exceed those of MPC-D 100/1127 in ventral depth (Figure 1 and 2, Table 1).

## Discussion

The dissimilarity among the anterior chevrons of MPC-D 100/1002 and MPC-D 100/1127 cannot be readily explained as pathologic. In both specimens, the chevrons show no signs of rugosities or of bilateral asymmetries, and neither form is limited to a single chevron. The finger-like form of MPC-D 100/1127 is shared by all anterior haemal spines, and the more unusual spear-head haemal spine form of MPC-D 100/1002 manifests progressively across the second, third, and fourth haemal spines (Figure 1 and 2). Similarly, the differences between the haemal spine forms of MPC-D 100/1002 and MPC-D 100/1127 are too extreme and purposive to be reasonably attributed to the degree of individual variation that is expected among con-specifics.

The possibility that the observed discrepancies in chevron form are the result of sexual dimorphism merits consideration. It has been previously reported that the anterior chevrons of modern crocodilians are sexually dimorphic^9^,^10^,^11^,^12^,^13^, and it has been previously hypothesized and widely repeated within the literature that the anterior chevrons of non-avian theropod dinosaurs were as well^10^,^11^,^12^,^13^,^14^,^15^. Two functional explanations have been offered to explain alleged sex-specific chevron forms. First, anterior chevrons with reduced haemal spines would theoretically increase the space between the axial skeleton and the posterior projection of the ischium. This would provide more room for the oviduct and for the passage of eggs^12^,^13^. Thus, reduced anterior haemal spines would be a female characteristic. Second, the haemal spine of the first chevron could serve as an attachment surface for the penis retractor muscle^10^,^11^,^13^. Following this explanation, lengthy anterior haemal spines would be a characteristic of males, which would benefit from the potentially larger surface for muscle attachment. Oviraptorosaurs are known to have laid eggs that were large in comparison to adult body size and to have laid pairs of eggs simultaneously^16^,^17^,^18^,^19^. Female oviraptorosaurs were therefore particularly likely to have had large pelvic canals, which would make oviraptorosaurs good candidates to display sexually dimorphic chevrons. However, in a reconsideration of chevron sexual dimorphism in crocodilians, Erickson et al.^20^ examined the morphology of 36 *Alligator mississippiensis* of known sex and found no significant support for the claim that chevron shape is a means of determining sex. Similarly, Peterman and Gauthier^21^ recently reported no evidence of chevron sexual dimorphism in a survey of 31 specimens of the Tiger Whiptail lizard (*Aspidoscelis tigris*). These results cast serious doubt on expectations of identifying sexually dimorphic chevrons in non-avian theropods for the previously hypothesized reasons.

More recently, based on numerous anatomical traits related to enhanced caudal musculature and caudal flexibility and on the discovery of feather-fan supporting pygostyles in multiple oviraptorosaur genera, Persons et al.^7^ offered the hypothesis that oviraptorosaurs had tails that were uniquely adapted to serve as dynamic display structures (this function appears to be supported by reconstructed evolutionary changes in intervertebral joint stiffness^22^). Persons et al.^7^ postulated that these caudal displays were likely employed during courtship rituals. Following this, Persons et al.^7^, predicted that oviraptorosaur tails would be found to show sexual dimorphism. It is not clear how all aspects of the chevron forms of MPC-D 100/1002 and MPC-D 100/1127 might enhance the theoretical role of the tail as a display structure. However, the anterior and posterior projections of the haemal spines of MPC-D 100/1002 certainly increase the surface area available for caudal muscle insertions. Furthermore, the relatively expanded ventral haemal tips of MPC-D 100/1002 provided enlarged insertion surfaces for the m. ischiocaudalis^23^, which is a key muscle in controlling lateral and ventral tail flexure.

If sexual dimorphism is accepted as an explanation for the morphological differences between MPC-D 100/1002 and MPC-D 100/1127, then the question of which of the two forms represents which sex remains. Based on the results from the comparative studies of modern crocodilian and lacertian dimorphism, it appears that chevron form is not a common and generally reliable indicator of sex. However, if it is true that shorter anterior haemal spines are an adaptation that facilitates enlarged oviduct size^12^,^13^, then the short haemal spines of MPC-D 100/1127 would be regarded as the female characteristic and the longer haemal spines of MPC-D 100/1002 would be regarded as the male character. Similarly, if a longer and more robust first chevron does facilitate anchoring of the penis retractor muscle^10^,^11^,^12^, then the longer and broader tipped haemal spines of MPC-D 100/1002 is the male character and the shorter and slighter haemal spines of MPC-D 100/1127 would be regarded as the female form. Lastly, if it true that oviraptorosaur tails were specialized to serve as dynamic display structures and haemal spines with increased surface areas for muscle insertion facilitated such displays^7^, the longer and broader tipped haemal spines of MPC-D 100/1002 would be regarded as the male form. This is suggested because gaudy feather-fanning courtship and other social displays are typically performed by males among modern birds (e.g. peafowl, sage grouse, turkeys etc.)^24^,^25^,^26^. Thus, regardless of which of the functional interpretations is/are considered correct, MPC-D 100/1002 is most probably male, and MPC-D 100/1127 is most probably female.

Finally, it should be noted that the possible instance of sexual dimorphism described is, at present, limited to *Khaan mckennai* and is unrecognized in any other species of oviraptorosaurs. Examination of the well preserved axial skeletons of eight individuals of *Conchoraptor gracilis* (MPC-D 102/3, and a suite of casts of poached specimens – University of Alberta Laboratory for Vertebrate Paleontology UALVP 54983, 54984, 54986, and 54987) reveals no strong variation in the form of their anterior chevrons (see Figure 2 above and Table 1 in supplementary information). These *Conchoraptor gracilis* specimens are largely articulated and part of a single bonebed layer, with a taphonomic history that is presumably similar to that of MPC-D 100/1127 and MPC-D 100/1002. In all cases, the *Conchoraptor gracilis* chevrons are similar in general shape to those of MPC-D 100/1127, with no specimens showing the spear-head form of MPC-D 100/1002. Social display structures frequently vary between closely related species, while the gross anatomy of internal reproductive structures generally does not. Thus, if it is true that not all genera of oviraptorosaurs were sexually dimorphic in anterior-chevron form, then this interspecific discrepancy supports the interpretation of the observed dimorphism in *Khaan mckennai* as functioning to facilitate social displays. Other oviraptorosaurs are known from too few specimens to allow for similar consideration. However, articulated skeletons of oviraptorosaurs are more common than those of most other non-avian theropods. The recognition of the dimorphism observed in the anterior chevrons of *Khaan mckennai* will hopefully inspire similar close observations among other oviraptorid species as they are collected, so that the extent of the dimorphism may be better established within the group.

## Author Contributions

W.S.P.IV, P.J.C. and M.A.N. wrote the main manuscript text. W.S.P.IV prepared Figure 1, and W.S.P.IV and G.F.F. prepared Figure 2. All authors reviewed the manuscript.

## Supplementary Material

Supplementary Information Supplementary Information

## Figures and Tables

**Table 1:** Caudal vertebra and chevron measurements of MPC-D 100/1002 and MPC-D 100/1127 -- “???” indicates measurements that were obscured and could not be reliably measured

MPC-D 100/1002						
	1st caudal	2nd caudal	3rd caudal	4th caudal	5th caudal	6th caudal
Centrum length across dorsal surface	16 mm	18 mm	18 mm	18 mm	17 mm	17 mm
Anterior centrum height	16 mm	15 mm	???	11 mm	???	???
Neural spine height above centrum	19 mm	18 mm	17 mm	???	???	???
Caudal rib lateral width	30 mm	29 mm	33 mm	31 mm	31 mm	31 mm

	1st chevron	2nd chevron	3rd chevron	4th chevron
Caudal articulation	2nd,3rd	3rd,4th	4th,5th	5th,6th
Dorsoventral depth	51 mm	60 mm	51 mm	50 mm
Ventral-tip anteroposterior length	10 mm	11 mm	13 mm	15 mm

MPC-D 100/1127						
	1st caudal	2nd caudal	3rd caudal	4th caudal	5th caudal	6th caudal
Centrum length across dorsal surface	15 mm	18 mm	18 mm	18 mm	17 mm	17 mm
Anterior centrum height	15 mm	14 mm	11 mm	???	???	???
Neural spine height above centrum	19 mm	18 mm	16 mm	15 mm	13 mm	12 mm
Caudal rib lateral width	23 mm	21 mm	27 mm	24 mm	24 mm	22 mm

	1st chevron	2nd chevron	3rd chevron	4th chevron
Caudal articulation	2nd,3rd	3rd,4th	4th,5th	5th,6th
Dorsoventral depth	45 mm	52 mm	43 mm	39 mm
Ventral-tip anteroposterior length	4 mm	7 mm	7 mm	6 mm
