# Supplementary material for: A possible instance of sexual dimorphism in the tails of two oviraptorosaur dinosaurs
Source: Sci Rep. 2015 Mar 31;5:9472. doi: 10.1038/srep09472 (PMC4379468; doi:10.1038/srep09472)
Supplement: Supplementary Information [file srep09472-s1.pdf]

A possible instance of sexual dimorphism in the tails of two oviraptorosaur dinosaurs

W. Scott Persons IV[1*], Gregory F. Funston[1], Philip J. Currie[1], and Mark A. Norell[2]

[1]*University of Alberta, Department of Biological Sciences, Edmonton, Alberta, T6G2E9, Canada;*
[2]*American Museum of Natural History, New York City, New York, USA.*
**Correspondence to persons@ualberta.ca*

UALVP 54983-1

	1st caudal	2nd caudal	3rd caudal	4th caudal	5th caudal	6th caudal
Centrum length across dorsal surface	23 mm	???	19 mm	20 mm	18 mm	18 mm
Anterior centrum height	19 mm	???	16 mm	14 mm	???	13 mm
Neural spine height above centrum	???	???	???	???	???	???
Caudal rib lateral width	24 mm	???	???	???	???	???

	1st chevron	2nd chevron	3rd chevron	4th chevron
Caudal articulation	2nd, 3rd	3rd, 4th	4th, 5th	5th, 6th
Dorsoventral depth	59 mm	72 mm	68 mm	64 mm
Heamal arch length	9 mm	10 mm	14 mm	14 mm
Ventral-tip anteroposterior length	8 mm	8 mm	7 mm	7 mm

UALVP 54983-2

	1st caudal	2nd caudal	3rd caudal	4th caudal	5th caudal	6th caudal
Centrum length across dorsal surface	???	???	20 mm	19 mm	18 mm	18 mm
Anterior centrum height	???	20 mm	19 mm	18 mm	16 mm	16 mm
Neural spine height above centrum	???	???	???	???	???	???
Caudal rib lateral width	???	21 mm	???	32 mm	29 mm	???

	1st chevron	2nd chevron	3rd chevron	4th chevron
Caudal articulation	2nd, 3rd	3rd, 4th	4th, 5th	5th, 6th
Dorsoventral depth	57 mm	65 mm	72 mm	70 mm
Heamal arch length	8 mm	11 mm	???	12 mm
Ventral-tip anteroposterior length	5 mm	7 mm	6 mm	6 mm

UALVP 54983-3

	1st caudal	2nd caudal	3rd caudal	4th caudal	5th caudal	6th caudal
Centrum length across dorsal surface	???	???	???	24 mm	24 mm	24 mm
Anterior centrum height	???	???	???	19 mm	19 mm	18 mm
Neural spine height above centrum	???	???	???	???	???	???
Caudal rib lateral width	???	???	???	???	???	???

1st chevron	2nd chevron	3rd chevron	4th chevron

Caudal articulation	2nd, 3rd	3rd, 4th	4th, 5th	5th, 6th
Dorsoventral depth	86 mm	92 mm	80 mm	78 mm
Heamal arch length	???	12 mm	12 mm	16 mm
Ventral-tip anteroposterior length	10 mm	11 mm	10 mm	9 mm

UALVP 54984

	1st caudal	2nd caudal	3rd caudal	4th caudal	5th caudal	6th caudal
Centrum length across dorsal surface	???	23 mm	23 mm	23 mm	25 mm	23 mm
Anterior centrum height	???	???	???	???	???	???
Neural spine height above centrum	???	???	???	???	???	???
Caudal rib lateral width	16 mm	16 mm	29 mm	29 mm	27 mm	19 mm

	1st chevron	2nd chevron	3rd chevron	4th chevron
Caudal articulation	2nd, 3rd	3rd, 4th	4th, 5th	5th, 6th
Dorsoventral depth	73 mm	70 mm	65 mm	66 mm
Heamal arch length	14 mm	13 mm	12 mm	11 mm
Ventral-tip anteroposterior length	8 mm	8 mm	7 mm	7 mm

UALVP 54986

	1st caudal	2nd caudal	3rd caudal	4th caudal	5th caudal	6th caudal
Centrum length across dorsal surface	19 mm	18 mm	18 mm	18 mm	17 mm	17 mm
Anterior centrum height	???	17 mm	16 mm	15 mm	13 mm	13 mm
Neural spine height above centrum	???	???	???	???	???	???
Caudal rib lateral width	15 mm	21 mm	25 mm	27 mm	24 mm	20 mm

	1st chevron	2nd chevron	3rd chevron	4th chevron
Caudal articulation	2nd, 3rd	3rd, 4th	4th, 5th	5th, 6th
Dorsoventral depth	52 mm	53 mm	58 mm	53 mm
Heamal arch length	8 mm	9 mm	10 mm	6 mm
Ventral-tip anteroposterior length	6 mm	8 mm	7 mm	7 mm

UALVP 54987

	1st caudal	2nd caudal	3rd caudal	4th caudal	5th caudal	6th caudal
Centrum length across dorsal surface	???	20 mm	23 mm	21 mm	24 mm	23 mm

Anterior centrum height	???	???	???	19 mm	19 mm	17 mm
Neural spine height above centrum	???	???	???	36 mm	31 mm	26 mm
Caudal rib lateral width	27 mm	23 mm	30 mm	30 mm	26 mm	22 mm

	1st chevron	2nd chevron	3rd chevron	4th chevron
Caudal articulation	2nd, 3rd	3rd, 4th	4th, 5th	5th, 6th
Dorsoventral depth	???	62 mm	63 mm	69 mm
Heamal arch length	???	12 mm	12 mm	11 mm
Ventral-tip anteroposterior length	???	8 mm	7 mm	7 mm

UALVP 54988

	1st caudal	2nd caudal	3rd caudal	4th caudal	5th caudal	6th caudal
Centrum length across dorsal surface	26 mm	???	22 mm	21 mm	20 mm	19 mm
Anterior centrum height	11 mm	???	???	???	???	???
Neural spine height above centrum	25 mm	21 mm	???	23 mm	20 mm	18 mm
Caudal rib lateral width	???	34 mm	33 mm	33 mm	26 mm	25 mm

	1st chevron	2nd chevron	3rd chevron	4th chevron
Caudal articulation	2nd, 3rd	3rd, 4th	4th, 5th	5th, 6th
Dorsoventral depth	???	59 mm	56 mm	54 mm
Heamal arch length	???	9 mm	10 mm	10 mm
Ventral-tip anteroposterior length	???	9 mm	8 mm	8 mm

Supplementary Table 1. Caudal vertebra and chevron measurements of seven specimens of *Conchoraptor gracilis* -- "???" indicates measurements that were obscured and could not be reliably measured.
